# The role of hyperbaric oxygen therapy in Fournier’s Gangrene: A systematic review and meta-analysis of observational studies

**DOI:** 10.1590/S1677-5538.IBJU.2022.0119

**Published:** 2022-05-10

**Authors:** Muhammad Achdiar Raizandha, Furqan Hidayatullah, Yudhistira Pradnyan Kloping, Ilham Akbar Rahman, Wahjoe Djatisoesanto, Fikri Rizaldi

**Affiliations:** 1 Universitas Airlangga Universitas Airlangga Teaching Hospital Faculty of Medicine Surabaya East Java Indonesia Department of Urology, Faculty of Medicine, Universitas Airlangga, Universitas Airlangga Teaching Hospital, Surabaya, East Java, Indonesia

**Keywords:** Fournier Gangrene, Hyperbaric Oxygenation, Debridement

## Abstract

**Purpose:**

Management of Fournier’s Gangrene (FG) includes broad-spectrum antibiotics with adequate surgical debridement, which should be performed within the first 24 hours of onset. However, this treatment may cause significant loss of tissue and may delay healing with the presence of ischemia. Hyperbaric oxygen therapy (HBOT) has been proposed as adjunctive therapy to assist the healing process. However, its benefit is still debatable. Therefore, this systematic review and meta-analysis aimed to evaluate the effect of HBOT as an adjunct therapy for FG.

**Materials and Methods:**

This study complied with the Preferred Reporting Items for Systematic Reviews and Meta-analyses protocol to obtain studies investigating the effect of HBOT on patients with FG. The search is systematically carried out on different databases such as MEDLINE, Embase, and Scopus based on population, intervention, control, and outcomes criteria. A total of 10 articles were retrieved for qualitative and quantitative analysis.

**Results:**

There was a significant difference in mortality as patients with FG who received HBOT had a lower number of deaths compared to patients who received conventional therapy (Odds Ratio 0.29; 95% CI 0.12 – 0.69; p = 0.005). However, the mean length of stay with Mean Difference (MD) of -0.18 (95% CI: -7.68 – 7.33; p=0.96) and the number of debridement procedures (MD 1.33; 95% CI: -0.58 – 3.23; p=0.17) were not significantly different.

**Conclusion:**

HBOT can be used as an adjunct therapy to prevent an increased risk of mortality in patients with FG.

## INTRODUCTION

Fournier’s Gangrene (FG) is a progressive infectious disease marked by necrotizing fasciitis of the perineum and external genitalia (1, 2). It is considered an emergency in Urology due to its tendency to develop into a severe soft tissue infection associated with systemic sepsis. In several cases, it also required amputation of the penis (3). FG mortality rate ranges from 18 to 50%, with an average of 20 to 30% (4). Management of FG includes aggressive resuscitation, broad-spectrum antibiotics, and surgical debridement, which should be done in under 24 hours (5). Despite this current standard therapy, FG still causes high mortality. It is possibly due to poor local blood supply in FG patients, causing infection and damage to the blood vessels, thus may delay healing. Aggressive debridement, in this case, may cause significant loss of tissue which prolongs the healing process causing long hospital stays and a high mortality rate (6).

This problem leads to Hyperbaric Oxygen Therapy (HBOT) as adjunctive therapy for FG. Hyperbaric oxygen therapy (HBOT) is a therapeutic option involving inhaling pressurized 100% oxygen in sealed chamber (7). HBOT allows the speeding up of the healing process, which increases tissue oxygen tension, and inhibits and kills anaerobic bacteria. HBOT possessed a bactericidal effect on anaerobic infection due to aerobic or anaerobic bacteria. Recent studies have reported the role of HBOT in significantly decreasing mortality in Fournier Gangrene patients (8). There is no consensus regarding the role of adjunctive therapy of HBOT in FG, and it is still debated whether it can be used to manage FG (4, 9). Therefore, this study aims to evaluate the effect of HBOT as an adjunct therapy for FG.

## MATERIALS AND METHODS

This study was in accordance with the Preferred Reporting Items for Systematic Reviews and Meta-analyses (PRISMA) protocol. Preliminary searching was performed to ensure that the PICO characteristics were yet to be investigated and avoid duplication of meta-analysis. Literature searches were conducted through MEDLINE, EMBASE, and Scopus databases. Applied key words were specified as (“Fournier Gangrene” or “penile necrotizing fasciitis”) and (“hyperbaric oxygen” or “hyperbaric oxygen therapy” or “hyperbaric oxygen treatment”). The expanded searching terms are presented in Table-1. The protocol of this study was registered on PROSPERO (CRD42021283421).

**Table 1:** Systematic search using relevant keywords.

Database	Keywords	Articles (n)
PubMed/MEDLINE	((((Fournier Gangrene) OR (Penile necrotizing fasciitis)) AND (Hyperbaric oxygen)) OR (Hyperbaric oxygen therapy)) OR (Hyperbaric oxygen treatment)	90
Scopus	((((Fournier Gangrene) OR (Penile necrotizing fasciitis)) AND (Hyperbaric oxygen)) OR (Hyperbaric oxygen therapy)) OR (Hyperbaric oxygen treatment)	191
EMBASE	((((Fournier gangrene) OR (penile necrotizing fasciitis)) AND (hyperbaric oxygen)) OR (hyperbaric oxygen therapy)) OR (hyperbaric oxygen treatment)	173
	**TOTAL**	**454**

### Inclusion and Exclusion Criteria

Articles permitted for inclusion must have been a randomized controlled trial or observational research, written in English, comprising a minimum of two arms, reporting the number of debridement, length of stay, and mortality rates in patients with FG who were treated with HBOT as opposed to only conventional therapy. Experimental trials in animals, unpublished articles, and abstract-only findings were excluded. Hyperbaric oxygen therapy (HBOT) is an adjunctive treatment in which the patients inhale 100% O^2^ fraction while being exposed to rising atmospheric pressure. The interventional arm was compared to the standard conventional therapy without HBOT.

### Data Extraction

Two independent investigators retrieved the data according to the extraction template. Any discrepancies and disagreements regarding data extraction would be discussed and decided by a third investigator as needed. The extracted items included study characteristics (authors, time of publication, number of samples, study design, inclusion and exclusion criteria, duration of follow-up); baseline characteristics of the subjects (age, type of intervention, affected anatomical region, and location of the study); and quantitative outcomes (length of stay, number of debridement procedures, and number of deaths).

### Quality Assessment

The risk of research bias was assessed using The Newcastle-Ottawa Scale (NOS), including selection, comparability, and exposure parameters. This scoring system was used to assess the risk of bias in non-randomized studies. The result from NOS instrument assessment is classified into three categories. A score of 0-3 indicates a low-quality study, while 4-6 indicates a medium quality study, and 7-9 indicates a high-quality study.

### Statistical Analysis

The measured endpoints were the mean number of debridement, mean length of stay, and mortality rate. The dichotomous variable was analysed using Odds Ratio (OR) at 95% Confidence Interval (CI), with a p-value below 0.05 regarded as statistically significant. Secondary outcomes were measured as a continuous variable with Weighted Mean Difference (WMD). Analysis of heterogeneity between studies was calculated using I^2^. Heterogeneity is considered high if the I^2^ is above 50%. Subsequently the random-effects model will be applied for pooled analysis. Otherwise in I^2^ <50%, the statistical fixed-effects model will be used. Statistical analysis was performed using RevMan 5.4 for Windows software and presented in the form of forest plots and descriptive narratives.

## RESULTS

### Systematic search results

**Figure 1:**
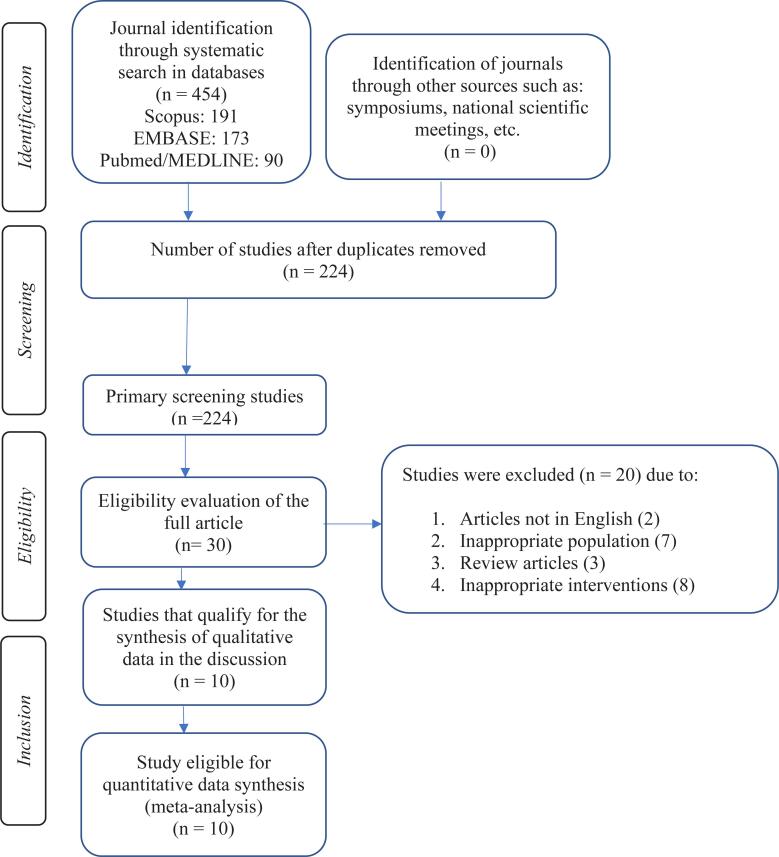
Study selection based on the PRISMA 2020 flowchart.

The initial search of the study database using specific key words (Table-1) yielded 454 studies. However, we removed 194 studies with irrelevant abstracts or titles and 230 duplicate studies. A total of 30 full-text studies were then assessed for eligibility. Finally, ten eligible studies were included in the analysis of this study (Figure-1).

### Baseline characteristics of the included studies

The characteristics of each included study are presented in Supplementary Table-1, which consists of the author and year of published studies, study design, description of the intervention, the mean age, comorbidities, and FGSI score. All included studies were retrospective studies that were published between 1998 and 2021. The total number of patients analysed in this meta-analysis was 657 patients consisting of 268 in the HBOT group and 369 in the non-HBOT group, with the average age of each study ranging from 46.13 to 68.3 years old. The intervention groups of each study were given a different dose of HBOT. However, only three studies mentioned the mean FGSI score of the included studies, ranging from 7.38 to 9 (10-12). Fournier gangrene patients were associated with several comorbidities such as diabetes, alcoholism, hypertension, and smoking. The assessed outcome of this study includes mortality, mean length of stay, and mean number of debridement, as described in Table-2.

**Table 2:** Evaluated Parameters in the assessment of the outcome.

Study (Years)	Study Type	Intervention	Sample Number	Outcome		
				Mortality (event/total)	Number of debridement (mean ± SD)	Length of stay (mean ± SD)
Feres et al.,2021 (7)	Retrospective	With Hyperbaric Oxygen Therapy	79	3 / 77	NR	NR
		Without Hyperbaric Oxygen Therapy	118	34 / 118	NR	NR
Creta et al.,2020 (10)	Retrospective	With Hyperbaric Oxygen Therapy	72	14 / 72	NR	NR
		Without Hyperbaric Oxygen Therapy	89	32 / 89	NR	NR
Tutino et al.,2020 (18)	Retrospective	With Hyperbaric Oxygen Therapy	13	2/13	NR	NR
		Without Hyperbaric Oxygen Therapy	10	1/10	NR	NR
Anheuser et al., 2018 (14)	Retrospective	With Hyperbaric Oxygen Therapy	17	0 / 17	13.3 ± 6.3	NR
		Without Hyperbaric Oxygen Therapy	45	2 / 45	4.8 ± 2.9	NR
Ferretti et al.,2017 (12)	Retrospective	With Hyperbaric Oxygen Therapy	3	0/3	NR	NR
		Without Hyperbaric Oxygen Therapy	16	3/16	NR	NR
Hung et al.,2016 (17)	Retrospective	With Hyperbaric Oxygen Therapy	12	0/12	NR	NR
		Without Hyperbaric Oxygen Therapy	48	32/48	NR	NR
Li et al.,2015 (11)	Retrospective	With Hyperbaric Oxygen Therapy	16	2 / 16	1.32 ± 0.48	31.4 ± 12.51
		Without Hyperbaric Oxygen Therapy	12	4 / 12	2.17 ± 0.72	31.3 ± 14.47
Mindrup et al.,2005 (13)	Retrospective	With Hyperbaric Oxygen Therapy	26	7 / 26	1.75 ± 0.878	30.8 ± 17
		Without Hyperbaric Oxygen Therapy	16	2 / 16	1.75 ±0.878	31.3 ± 18.2
Ayan et al.,2005 (15)	Retrospective	With Hyperbaric Oxygen Therapy	18	0/18	NR	NR
		Without Hyperbaric Oxygen Therapy	23	9/23	NR	NR
Hollabaugh et al., 1998 (16)	Retrospective	With Hyperbaric Oxygen Therapy	14	1/14	NR	NR
		Without Hyperbaric Oxygen Therapy	12	5/12	NR	NR

### Risk of bias assessment

We used the NOS instrument to assess the risk of bias in this meta-analysis. The result from the assessment using NOS instrument of the included studies ranged from 6 to 8 which indicates a moderate to a high-quality assessment of the risk of bias (Table-3).

**Table 3:** NOS instrument to assess the risk of bias of the study.

No.	Author	Year	Type of Studies	Quality Score			
				Selection	Comparability	Exposure	Total
1	Feres et al., (7)	2021	Retrospective study	3	1	3	7
2	Creta et al., (10)	2020	Retrospective study	3	2	3	8
3	Tutino et al., (18)	2020	Retrospective study	3	1	2	6
4	Anheuser et al., (14)	2018	Retrospective study	3	0	3	6
5	Ferretti et al., (12)	2017	Retrospective study	3	1	3	7
6	Hung et al., (17)	2016	Retrospective study	3	2	2	7
7	Li et al., (11)	2015	Retrospective study	4	0	2	6
8	Mindrup et al., (13)	2005	Retrospective study	3	0	3	6
9	Ayan et al., (15)	2005	Retrospective study	3	1	2	6
10	Hollabaugh et al., (16)	1998	Retrospective study	3	1	2	6

### Meta-analysis results on mortality

**Figure 2:**
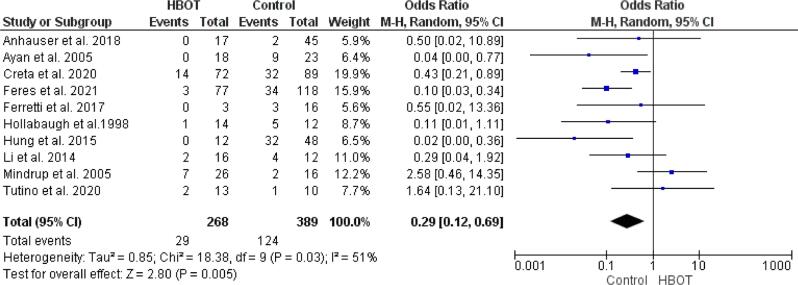
Forest plot for the mortality rate of FG patients in HBOT and non-HBOT groups.

Based on the analysis of ten included studies (7, 10-18), patients with HBOT have a significantly lower mortality rate than patients without HBOT (OR 0.29; 95%CI 0.12, 0.69; p=0.005) (Figure-2). The random-effects model was used due to high heterogeneity between studies (P = 0.03; I^2^ = 51%). Of the ten studies, Creta et al. and Feres et al. have notable significance in the analysis due to the larger number of samples compared to other studies (7, 10). Only two studies reported an increase in mortality in patients treated with HBOT (13, 18).

### Meta-analysis result on the length of stay

**Figure 3:**
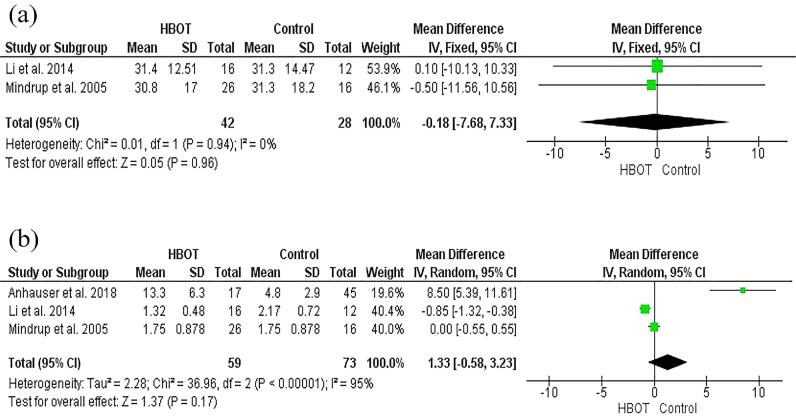
a) Forest plot for the length of stay of FG patients in HBOT and non-HBOT groups, b) Forest plot for the number of debridement of FG patients in HBOT and non-HBOT groups.

The forest plot analysis in this study also evaluated the difference in length of stay between HBOT and non-HBOT groups. The analysis results of two included studies (11, 13) did not reveal any significant difference regarding the mean length of stay between the HBOT and non-HBOT groups in FG patients (MD -0.18; 95%CI: -7.68 – 7.33; p=0.96) (Figure-3a). The fixed-effects model was used due to low heterogeneity between studies (p = 0.94; I^2^ = 0%).

### Meta-analysis results on the number of debridement

This meta-analysis also compared the number of debridement procedures performed in HBOT and non-HBOT groups. Three included studies (11, 13, 14) in the analysis of this outcome revealed no significant difference in the mean number of debridement procedures between HBOT and non-HBOT in FG patients (MD 1.33; 95% CI -0.58-3.23; p=0.17) (Figure-3b). The random-effects model was used due to the heterogeneity between studies was high at 95% (<0.00001; I^2^ 95%)

## DISCUSSION

To the best of our knowledge, this is the first systematic review and meta-analysis study on the evaluation of HBOT in Fournier Gangrene patients. Oxygen therapy (HBOT) is an adjunctive treatment to the primary surgical debridement in the cases of soft tissue infection. This treatment involves inhaling 100% fraction of Oxygen in a pressurized environment. However, the benefit of HBOT for Fournier Gangrene (FG) is still controversial (19). Further investigation is needed before HBOT can be recommended for routine use in cases of FG. Our study demonstrated a significant result that HBOT might reduce the mortality rate in FG patients. However, the effect of HBOT on the length of stay and number of debridement was not proven in this study.

Several previous studies have proven that the most important intervention to control the progressivity of the rapidly infectious process of FG involves repeated surgical debridement, broad-spectrum antibiotics, and intensive care. However, FG patients still posses a high risk of mortality and morbidity. Finding an adjunctive treatment to the standard treatment was crucial and may significantly benefit survival and prevent higher mortality of FG patients. This meta-analysis revealed a significantly lower mortality rate in FG patients who received adjuvant HBOT than conventional therapy (OR 0.29; 95% CI 0.12, 0.69; p = 0.005), consistent with findings in several studies (10, 11, 20, 21). A study by Anheuser et al. (2018) reported that this promising result in the HBOT group was also influenced by the well availability of hyperbaric oxygen therapy and safe patient transfer despite the patient’s poor physical condition because delaying the patient transfer to surgical debridement may significantly increase mortality rate (14). However, HBOT alone cannot replace the initial treatment of FG, which includes aggressive resuscitation, broad-spectrum antibiotic therapy, early colostomy, and adequate debridement (17). Another study suggested that HBOT became an independent predictor for decreased mortality rate due to Fournier Gangrene (12). A study by Mindrup et al. (2005) has contradictory results regarding the HBOT group’s mortality rate. It revealed that patients who underwent HBOT have a higher mortality rate, 12.5% in the non-HBOT group and 26.9% in the HBOT group (13). On the other hand, a study by Pizzorno et al. (1997) showed 0% mortality rate in patients that did not undergo HBOT (22), while other studies only reported a 3 and 9% mortality rate (23, 24). Differences may occur due to several factors which may affect mortality in the treatment of Fournier Gangrene patients, such as surgeon experience, early administration of antibiotic therapy, intensive care, and early surgical therapy (22, 25-27). Another study also reported that the surface area of the infected body is also a factor that affects survival and mortality (28). Hyperbaric oxygen therapy was considered to be safe because it did not cause a delay in surgical debridement or interrupt the standard therapy.

The length of stay between the two studies did not reveal a significant difference (MD -0.18; 95%CI: -7.68 – 7.33). Only one study reported a reduction in length of stay among patients with FG receiving HBOT (29). However, the sample of this study was consisted of HBOT and NPWT treatment thus it was difficult to confirm specifically the adjunctive effect of HBOT treatment in FG patients. According to a study by Anheuser et al., there was no difference in patients with FG receiving HBOT in terms of length of stay (14). Other study also reported a shorter length of stay along and decreased mortality rates (10). In relation to the length of stay, physical disability is a significant predictor of longer hospitalization (13). It could be due to community issues, as approximately 30% of FG patients require treatment at rehabilitation centres, long-term care facilities, or local hospitals (13). The length of stay was also influenced by the need to perform concurrent surgeries such as colostomy. Regarding Fournier Gangrene Severity Index score, sepsis significantly influences the length of stay in FG patients. Understanding the importance of predicting length of stay may provide strategy in patient-based treatment and aid in decision-making in treatment choice.

Pooled analysis of the number of debridement procedures suggested no significant difference between HBOT and conventional therapy (MD 1.33; 95% CI: -0.58 – 3.23). A previous study reported that the average number of surgical debridement procedures was similar between HBOT and conventional therapy leading to the interpretation that HBOT had no advantage in decreasing the number of debridement procedures when used as an adjuvant treatment of FG (30). A lower number of debridement procedures among control that did not receive HBOT has also been reported. The number of required debridement was an important parameter because complete recovery in FG patients may be determined with a lower number of repeated debridement (11).

Based on a study reported by Mindrup et al., the cost of HBOT was not negligible, as hospital charges were significantly higher among HBOT group (13). A study conducted in Germany stated that the availability of HBOT was relatively low. In addition, the expense of a patient treated with the HBOT ranges from 8,000 to 25,000 EUR and is not covered by health insurance (14). Therefore, the recommendations of HBOT as adjunctive therapy requires more cost analysis studies before it can be implemented for routine use in FG cases.

Several limitations existed in this study. Firstly, other factors that may affect the outcome cannot be entirely analysed, leaving the possibility of influence on the outcome results. Secondly, cost analysis could not yet be performed as only a few included studies mentioned this aspect in relation to the given intervention. Thirdly, the high heterogeneity of the included studies occurred due to various characteristics among study population, including patient comorbidities in both arms, the manner of the intervention, and the endpoint for analysis. Therefore, it is necessary to conduct research with a uniform design setting and population. Lastly, all included studies were retrospective observational studies. The nature of this design may raise several biases. More studies on this topic should be done, especially randomized-control trial studies, to create an adequate analysis of the usage of Hyperbaric Oxygen for Fournier’s Gangrene Patients.

## CONCLUSION

The adjunctive therapy of Hyperbaric Oxygen possessed a significantly lower mortality rate compared to conventional therapy. However, the effect of HBOT on the length of stay and number of debridement was not proven in this study. The influence of multiple factors warrants the need for future randomized controlled trials.

## ABBREVIATIONS

HBOT: Hyperbaric Oxygen Therapy
FG: Fournier’s Gangrene
PRISMA: Preferred Reporting Items for Systematic Review and Meta-Analyses
PICO: Population-Intervention-Comparison-Outcome
MD: Mean Difference
OR: Odds Ratio
CI: Confidence Interval
NOS: Newcastle-Ottawa Scale
WMD: Weighted Mean Difference
FGSI: Fournier’s Gangrene Severity Index

## APPENDIX:

[Table t4]

**Supplementary Table 1 t4:** Baseline characteristics of the included studies.

Study(Years)	Study Type	Group	Age(mean)	Type ofIntervention	Comorbidity				FGSI Score (mean ± SD)
					Diabetes Mellitus (n, %)	Alcoholism (n, %)	Hypertension (n, %)	Smoking (n, %)	
Feres et al., 2021 (7)	Retrospective	With Hyperbaric Oxygen Therapy	48.2 (10 – 81)	HBOT was administered once daily for a total of 15 sessions (2.4 atmospheric oxygen, for 2 hours)	29 (36.7)	28 (35.4)	27 (34.1)	24 (30.3)	NR
		Without Hyperbaric Oxygen Therapy	46.6 (1 – 82)		33 (27.9)	35 (29.6)	33 (27.9)	30 (25.4)	NR
Creta et al., 2020 (10)	Retrospective	With Hyperbaric Oxygen Therapy	64.3 (16.1)	HBOT given twice a day for 4-7 days (2.4 – 2.8 atmospheric oxygen, for 50 – 90 minutes)	NR	NR	NR	NR	9.0 ± 4.8
		Without Hyperbaric Oxygen Therapy	68.3 (14.2)		NR	NR	NR	NR	8.0 ± 4.0
Tutino et al., 2020 (18)	Retrospective	With Hyperbaric Oxygen Therapy	NR	HBOT was offered to 13 (56.5%) patients using a scheduled session of 60 minutes daily.	11 (55)	4 (15)	NR	11 (50)	NR
		Without Hyperbaric Oxygen Therapy							
Anheuser et al., 2018 (14)	Retrospective	With Hyperbaric Oxygen Therapy	58	NR	9 (52.9)	5 (29.4)	NR	NR	NR
		Without Hyperbaric Oxygen Therapy		60	23 (50)	14 (31.1)	NR	NR	NR
Ferretti et al., 2017 (12)	Retrospective	With Hyperbaric Oxygen Therapy	56 (19-74)	NR	3(16)	NR	NR	0 (0)	4.3 (3-6)
		Without Hyperbaric Oxygen Therapy			10 (53)			3(16)	8.3 (2-19)
Hung et al., 2016 (17)	Retrospective	With Hyperbaric Oxygen Therapy	59.6 ± 14.5	NR	44(73)	NR	25 (41)	NR	NR
		Without Hyperbaric Oxygen Therapy							
Li et al., 2015 (11)	Retrospective	With Hyperbaric Oxygen Therapy	46.13±13.11	HBOT given twice daily for 5-7 days (2.5 units of atmospheric oxygen, 90-120 minutes per session at 10 hours intervals)	NR	NR	NR	NR	7.38±3.20
		Without Hyperbaric Oxygen Therapy	48.42±15.31		NR	NR	NR	NR	7.42±3.20
Mindrup et al., 2005 (13)	Retrospective	With Hyperbaric Oxygen Therapy	57 ±14	HBOT given 1-3 times (2.4-3 units of atmospheric oxygen, 30-90 minutes)	19 (73)	6 (23)	4 (15)	19 (73)	NR
		Without Hyperbaric Oxygen Therapy	57 ±15		10 (63)	5 (31)	2 (13)	10 (63)	NR
Ayan et al., 2005 (15)	Retrospective	With Hyperbaric Oxygen Therapy	57.3 (35-74)	Hyperbaric Oxygen (HBO) using 2.5 atmospheric pressure for 90 min/day was applied to 18 patients (43.9%) for three to 10 days.	17 (41.4)	5 (12.1)	NR	NR	NR
		Without Hyperbaric Oxygen Therapy							
Hollabaugh et al., 1998 (16)	Retrospective	With Hyperbaric Oxygen Therapy	57 (26-87)	HBOT was offered to 14 (54%) patients, The dive parameters were 90 min at 2.4 atmospheres absolute/45 feet sea water. Duration of therapy averaged 12 days.	10 (38)	9 (35)	NR	NR	NR
		Without Hyperbaric Oxygen Therapy							
